# Orchestration of Immunological Synapse Assembly by Vesicular Trafficking

**DOI:** 10.3389/fcell.2019.00110

**Published:** 2019-07-03

**Authors:** Anna Onnis, Cosima T. Baldari

**Affiliations:** Department of Life Sciences, University of Siena, Siena, Italy

**Keywords:** TCR signaling, immune synapse, endocytosis, polarized recycling, vesicular trafficking

## Abstract

Ligation of the T-cell antigen receptor (TCR) by cognate peptide bound to the Major Histocompatibility Complex on the surface of an antigen-presenting cell (APC) leads to the spatial reorganization of the TCR and accessory receptors to form a specialized area of intimate contact between T cell and APC, known as the immunological synapse (IS), where signals are deciphered, coordinated, and integrated to promote T cell activation. With the discovery that an endosomal TCR pool contributes to IS assembly and function by undergoing polarized recycling to the IS, recent years have witnessed a shift from a plasma membrane-centric view of the IS to the vesicular trafficking events that occur at this location following the TCR-dependent translocation of the centrosome toward the synaptic membrane. Here we will summarize our current understanding of the trafficking pathways that are responsible for the steady delivery of endosomal TCRs, kinases, and adapters to the IS to sustain signaling, as well as of the endocytic pathways responsible for signal termination. We will also discuss recent evidence highlighting a role for endosomes in sustaining TCR signaling after its internalization at the IS and identifying the IS as a site of formation and release of extracellular vesicles that allow for transcellular communication with the APC.

## Introduction

The differentiation of naïve T cells into armed effectors able to promote the elimination of pathogens or to kill cancer cells is initiated by signals triggered by the T-cell receptor (TCR) following engagement by specific peptide antigen associated to the Major Histocompatibility Complex (pMHC) on antigen-presenting cells (APC). These signals promote the spatiotemporally regulated assembly of a specialized membrane structure at the T cell-APC interface, known as the immunological synapse (IS). There TCRs, integrins, and co-stimulatory receptors accumulate and the respective signals are integrated to elicit both short-term effects such as the dynamic reorganization of the actin and tubulin cytoskeleton, and the initiation of a gene expression cascade that will ultimately lead to the generation of effector as well as memory T cells (Dustin and Choudhuri, 2016).

While the IS has long been considered from a plasma membrane-centric viewpoint, endocytic traffic has emerged as a central determinant in sustaining signaling at the IS over the extended timeframe required for T cell activation (Finetti et al., 2015a, 2017; Onnis et al., 2016). This function is mediated by an intracellular TCR pool associated with recycling endosomes that undergoes polarized delivery to the synaptic membrane to maintain a steady supply of receptors as engaged receptors undergo activation-dependent internalization (Soares et al., 2013a). Interestingly, TCR endocytosis does not only subserve the canonical function of terminating signaling through receptor downregulation but is also exploited to sustain signaling at the IS by making space for new, signaling-competent TCRs. Additionally, recent developments have highlighted new roles for endocytic traffic in IS signaling both inside the cell, where internalized TCRs sustain signaling from an endosomal localization, and at the IS membrane, wherefrom miRNA-enriched exosomes and TCR-enriched ectosomes are released for transcellular communication with the cognate APC (Mittelbrunn et al., 2011; Choudhuri et al., 2014). Here we will summarize our current understanding of endocytic traffic in the regulation of IS assembly and function. For the specific topic of exocytic traffic at the lytic synapse in cytotoxic T cells the reader is referred to the excellent review by Dieckmann et al. (2016).

## A Bird’s View of the Immune Synapse

Kupfer’s group described for the first time in the 1990s the contact area between T cell and cognate APC as a highly organized “bull’s eye” structure, known as the IS, with radially symmetric compartments, referred to as supramolecular activation clusters (SMAC) (Monks et al., 1998). The TCR accumulates in the central area of this structure, the central SMAC (cSMAC). The cSMAC is surrounded by an adhesive ring, the peripheral SMAC (pSMAC), where the integrin LFA-1 segregates, and a distal ring, the distal SMAC (dSMAC), that includes the transmembrane tyrosine phosphatase CD45 and dynamic actin (Freiberg et al., 2002). Although this particular architecture of the IS does not apply to all to T cell: APC contacts, and particularly to the interface of T cells with dendritic cells (DCs), which features multifocal synapses (Brossard et al., 2005; Thauland and Parker, 2010), our current understanding of the IS is largely based on the SMAC-based organization.

The development of powerful tools for live imaging has led to major revisions of Kupfer’s definition of SMACs. TCR binding to pMHC has been shown to trigger the reorganization of downstream signaling molecules into micrometer- or submicrometer-sized clusters (Choudhuri and Dustin, 2010; Yokosuka and Saito, 2010; Hashimoto-Tane and Saito, 2016). These microclusters (MCs) were initially described by Krummel and Davis as small clusters of approximately 100 TCR molecules that accumulate at the center of the T cell-APC interface upon stimulation (Krummel et al., 2000; Krummel and Davis, 2002). TCR-MCs can be readily assembled upon TCR engagement through the coalescence of preexisting TCR nanoclusters which, similar to the kinase Lck or the adapter LAT, form “protein islands” at the plasma membrane (Schamel et al., 2005; Lillemeier et al., 2010; Crites et al., 2014; Roh et al., 2015). TCR-MCs also contain the co-stimulatory receptor CD28, as well as kinases and signaling adapters, including ZAP-70, LAT, SLP-76, PLC-γ, and the cytoskeleton-related molecules Nck and Vav (Bunnell et al., 2002; Choudhuri and Dustin, 2010).

Reconstitution methods based on fluid supported lipid bilayers have provided evidence that TCR-MCs form at the dSMAC and move centripetally through the pSMAC to the cSMAC, initially with the assistance of actin filaments undergoing retrograde flow and subsequently through dynein-mediated movement along the microtubules toward the centrosome that translocates beneath the cSMAC following TCR stimulation (Campi et al., 2005; Hashimoto-Tane et al., 2011). Surprisingly, active Lck is found at the dSMAC, but not at the cSMAC (Lee et al., 2002). Additionally, neither CD28 nor the signaling molecules associated with TCR-MCs accumulate at the cSMAC together with the TCR. Rather, CD28 segregates to the TCR-poor border of the cSMAC (exo-cSMAC versus endo-cSMAC, corresponding to the TCR-rich central part of the cSMAC), where it assembles kinases to sustain signaling (Yokosuka et al., 2008). Hence the notion of the cSMAC as a structure that allows for sustained TCR signaling has been replaced with one which posits that signaling is initiated at TCR-MCs at the dSMAC, with the cSMAC functioning as a “sink” to remove TCRs that have become signaling-incompetent during their journey from the dSMAC.

As an exception to this scenario, the cSMAC actually acts as platform for sustained signaling when TCRs are engaged by weak pMHC ligands (Cemerski et al., 2008). Interestingly, TCR-MCs have recently been shown to be surrounded by a ring of LFA-1 associated at the cytosolic face with focal adhesion molecules (Hashimoto-Tane et al., 2016). These “microsynapses,” that disappear before the TCR-MCs move to the cSMAC, are important to ensure adhesion, which is a critical determinant for sustained TCR signaling, particularly when engaged by low affinity ligands.

## An Endocytic View of the Immune Synapse

The studies on IS assembly and function have largely dealt with events that occur at the plasma membrane, with a focus on the dynamics and segregation of TCRs, integrins and costimulatory molecules together with their associated signaling mediators during IS maturation. A major breakthrough came with the finding that the TCRs that accumulate at the cSMAC do not originate solely from the pre-existing plasma membrane pool, which would become exhausted during the prolonged timeframe of T cell activation as the result of the continuous endocytosis of engaged as well as bystander TCRs (Niedergang et al., 1997; Bonefeld et al., 2003; Monjas et al., 2004) at the cSMAC center. An intracellular pool of TCRs, associated with recycling endosomes, has been shown to play a key role in replenishing the plasma membrane pool through polarized recycling to the IS membrane (Das et al., 2004). Remarkably, other membrane-associated signaling mediators that are recruited to the IS, including Lck and LAT, exist as two pools, with the endosomal pool undergoing polarized recycling to the IS (Ehrlich et al., 2002; Bonello et al., 2004). In the next section we will review rapidly accumulating evidence for a role of endosome recycling in IS assembly and function, as well as the distinct trafficking pathways that specifically control this process for individual molecules.

### Polarized Endosome Recycling to the IS Sustains Signaling

At variance with effector T cells, and in particular cytotoxic T cells, which are readily activated upon TCR engagement even in the presence of few cognate pMHC (Purbhoo et al., 2004), naïve T cells require signaling to be sustained for several hours to become activated (Iezzi et al., 1998). Since engaged TCRs are rapidly downregulated, sustained signaling relies on the delivery of TCRs from the endosomal pool, which is mobilized to the centrosome that has translocated to the IS to undergo polarized recycling (Das et al., 2004). This process is also exploited to supply the IS with key components of the TCR signaling pathway, including the initiating kinase Lck and the adaptor LAT, which is essential to propagate TCR signaling. In this section we will review the trafficking pathways that orchestrate endosome recycling to the IS.

#### TCR Endocytosis

The TCR is a protein complex formed by an antigen-recognition module consisting of the α and β chains, and a signal transducing module consisting of a ζ-chain homodimer and four cluster of differentiation 3 (CD3) chains present as γε and δε heterodimers. The intracellular domains of the ζ-chains and each of the CD3 chains contain immunoreceptor tyrosine-based activation motifs (ITAMs) that allow for the recruitment of the intracellular signal transduction machinery upon TCR engagement (Courtney et al., 2018).

The ability of a T cell to become activated is critically regulated by the number of TCRs expressed on the plasma membrane. In unstimulated T cells, the levels of surface TCR depend on the fine balance of multiple processes, namely *de novo* synthesis and transport of newly assembled receptors, endocytosis of surface TCR, recycling to the plasma membrane of internalized receptors and receptor degradation. Since the rates of *de novo* synthesis and constitutive degradation are low, endosomal recycling is the principal mechanism exploited by T cells to regulate their surface TCR expression. Additionally, the periodic transit of the TCR-CD3 complex inside the cell has been proposed as an opportunity of quality control of this long-lived receptor (Alcover and Alarcón, 2000; Geisler, 2004; Alcover et al., 2018).

T-cell receptor binding to pMHC on the surface of a cognate APC results in the rapid re-orientation of the centrosome, which polarizes toward the center of the T cell-APC interface through a process controlled by a complex signaling pathway and involving a major reorganization of the microtubule and actin cytoskeletons. This leads to the concomitant polarization of the Golgi apparatus and endosomal network toward the IS (Sancho et al., 2002). Alcover’s group provided the first evidence that endosomal TCRs undergo polarized recycling to the IS membrane, to which they are delivered through a SNARE-mediated fusion event (Das et al., 2004). Additionally, non-engaged, non-phosphorylated surface TCRs distal to the IS are internalized and re-routed to the IS through their interaction which β-arrestin, which is phosphorylated by PKC in response to signals triggered by engaged TCRs (Fernández-Arenas et al., 2014).

**FIGURE 1:**
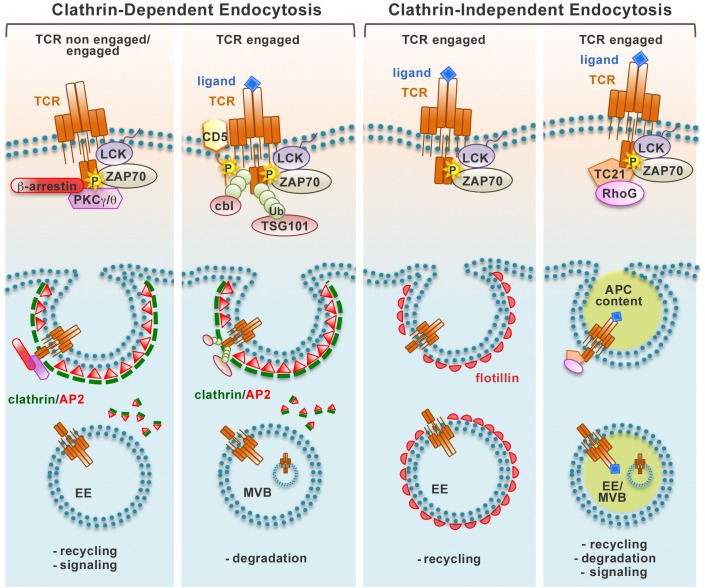
Pathways of TCR endocytosis. Non-engaged and ligand-engaged TCRs are internalized through different types of endocytic routes. The recruitment of different molecular players after TCR engagement induces the activation of distinct endocytic pathways that can be divided in two main groups, clathrin-dependent and clathrin-independent. Endocytic vesicles carrying TCRs internalized at the plasma membrane are incorporated into a network of endosomal compartments defined either by clathrin and the AP2 complex (clathrin-dependent endocytosis) or the membrane-organizing protein flotillins (clathrin-independent endocytosis). Alternatively, engaged TCRs are internalized together with bound pMHC trogocytosed from the APC through a clathrin-independent process. Internalized TCRs can be sorted to early endosomes (EE) and recycled to the plasma membrane through the recycling endosomes, remain associated to signaling endosomes, or traffic through late endosomes/multivesicular bodies (MVB) to the lysosomes for degradation.

The pathways that coordinate the different steps of TCR recycling have been elucidated only in part. Constitutive TCR endocytosis is dependent on the PKCα/PKCθ-mediated phosphorylation of residue S126 and on residues D127 and K128 located close to the di-leucine motif present on the intracellular domain of the CD3γ chain (Dietrich et al., 1994; von Essen et al., 2002), which has been proposed to allow for binding of the clathrin adaptor AP-2 and routing to the endosomal system (Dietrich et al., 1997). However, Szymczak and Vignali (2005) showed that the ITAMs are also required for AP-2 recruitment, with a higher efficiency for the CD3δ chain, indicating that AP-2-dependent TCR endocytosis depends both on di-leucine-based signals and on tyrosine-based signals. Clathrin-dependent endocytosis may be exploited not only for TCRs undergoing constitutive internalization in resting T cells (Dietrich et al., 1997; Schneider et al., 1999; Szymczak and Vignali., 2005) but also for the protein tyrosine kinase- and PKC/CD3γ-dependent internalization of bystander TCRs that are co-down-modulated with engaged TCRs during T cell activation (Monjas et al., 2004; Fernández-Arenas et al., 2014; Figure 1).

The signaling and trafficking pathways responsible of the internalization of engaged TCRs have been intensely debated. Both the CD3γ-regulated, PKC-dependent pathway and the ITAM-regulated, protein tyrosine kinase-dependent pathway have been implicated in this process (D’Oro et al., 1997; Lauritsen et al., 1998; Martin and Bevan, 1998; von Essen et al., 2002; Bonefeld et al., 2003; Crotzer et al., 2004), however others have ruled out the contribution of these pathways (Salio et al., 1997). Additionally, at variance with several reports indicating a role for clathrin-dependent endocytosis (Dietrich et al., 1994; von Essen et al., 2002; Crotzer et al., 2004), clathrin-independent endocytosis has been identified as the main pathway of internalization for engaged TCRs. Using a live imaging approach, Compeer et al. (2018) demonstrated that engaged surface TCRs are endocytosed through a clathrin-independent pathway and subsequently incorporated into a dynamic endocytic network defined by the membrane-organizing flotillins and essential for recycling of internalized TCRs and for early and late signaling events essential for T-cell activation. These results are in agreement with the finding that engaged TCRs are recruited to lipid rafts, which are known to be enriched in flotillins, and that these membrane microdomains are essential for their internalization (Monjas et al., 2004; Barr et al., 2006). A recent report has identified the small GTPase Rab22 and Arf6, a member of the ADP ribosylation factor family of GTP-binding proteins, as regulators of the clathrin-independent trafficking of LFA-1 and CD4, which are both important for conjugate formation (Johnson et al., 2017). Whether these proteins also regulate TCR recycling needs as yet to be elucidated. The participation of clathrin-dependent and -independent pathways could be reconciled by the finding that they are mainly operational at the pSMAC and the cSMAC, respectively (Martínez-Martín et al., 2011).

An additional, unusual clathrin-independent pathway of TCR downregulation at the IS has been described by Martínez-Martín et al. (2011) who provided evidence that engaged TCRs are internalized together with bound pMHC. This pathway is orchestrated by TC21/Rras2, a small GTPase that interacts constitutively with the TCR and is co-recruited to the IS, where it promotes TCR internalization through a mechanism involving the phagocytic GTPase RhoG (Martínez-Martín et al., 2011).

Independently of the pathway exploited for the internalization of engaged receptors, surface TCR expression is downregulated as a result of a shift toward decreased recycling and increased degradation of engaged receptors (Liu et al., 2000). Interestingly, T cells are able to discriminate between internalized engaged receptors and non-engaged bystander receptors, which leads to a preferential degradation of internalized engaged TCRs, while bystander co-internalized TCRs are predominantly recycled to the IS. An important determinant in the fate of internalized TCRs is likely to be ubiquitylation. TCR engagement results in the tyrosine kinase-dependent association of the ubiquitin ligases Cbl and Cbl-b with the inhibitory receptor CD5, leading to ubiquitylation of several substrates that include the TCR itself (Voisinne et al., 2016). Ubiquitylated TCR as well as its ubiquitylated signaling partners are recognized by TSG101, the ubiquitin-binding component of the ESCRT-I complex that controls receptor sorting into multivesicular bodies (MVB) (Vardhana et al., 2010). TSG101 accumulates at the cSMAC together with structures positive for the MVB marker lysobisphosphatidic acid and promotes TCR internalization, thereby providing an effective means to terminate signaling by TCRs that have been mobilized to the cSMAC from peripheral TCR-MCs (Vardhana et al., 2010; Figure 1).

How the engaged TCR are routed to a specific endocytosis pathway and the resulting outcome in terms of their subsequent fate is as yet an open question. The amount and/or affinity of ligand may be an important determinant in this choice (Figure 1).

#### TCR Recycling

Following internalization, plasma membrane receptors are delivered to early endosomes (EEs), which serve as a focal point of the endocytic pathway as they are responsible for sorting internalized cargo to either recycling endosomes (REs) for returning to the plasma membrane, or to late endosomes for lysosome-dependent degradation. Post-endocytic receptor traffic is orchestrated by ubiquitous Rab GTPases and their regulators and effectors that define the identity and function of the endosome subpopulations. The Rab GTPases localized at EEs that orchestrate recycling of internalized receptors are Rab5 and Rab4 (Pfeffer, 2013; Zhen and Stenmark, 2015). Coordination of cargo sorting with its fast, microtubule-independent recycling directly from EE is achieved by Rabenosyn-5, a FYVE-domain-containing Rab5 and Rab4 effector (Nielsen et al., 2000; De Renzis et al., 2002). Alternatively, internalized receptors undergo retrograde traffic to the perinuclear endocytic recycling compartment (ERC) to be incorporated into Rab11^+^ endosomes that are delivered to the plasma membrane through a slower, microtubule-dependent route (Zhen and Stenmark, 2015).

**FIGURE 2:**
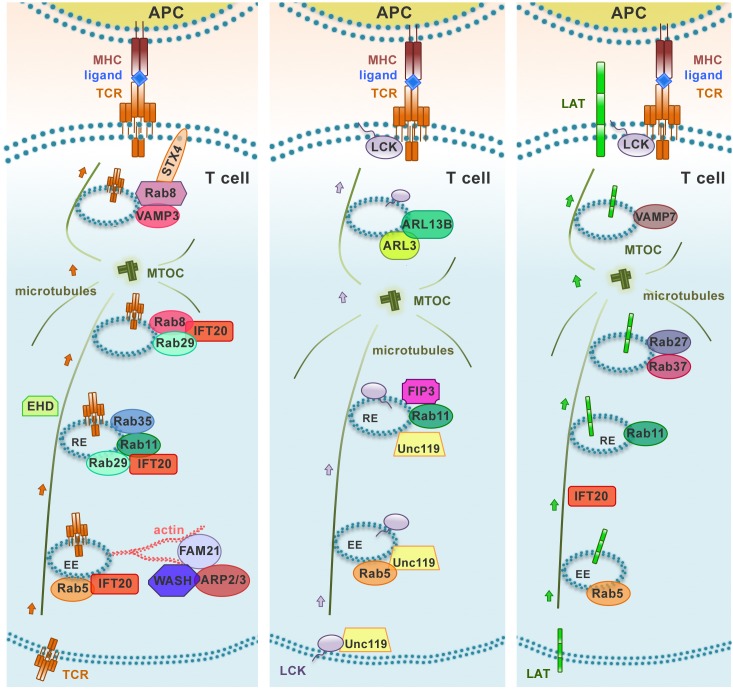
Distinct polarized recycling pathways for the TCR, LAT, and Lck. Upon TCR activation, TCR, LAT, and Lck are delivered from endosomal vesicles to the immune synapse through distinct trafficking routes. The distinct compartmentalization of these molecules within the endomembrane system is achieved through the combination of “classical” recycling Rab GTPases (Rab5 and Rab11) and individual trafficking regulators to achieve an efficient and accurate delivery to the immune synapse. EE, early endosome; RE, recycling endosome.

Similar to other recycling receptors, the TCR exploits these universal pathways to return to the plasma membrane. However, accumulating evidence highlights the existence of a highly regulated, tailor-made trafficking pathway that controls TCR recycling. Although this pathway has been studied mainly in the context of IS assembly and function following the discovery of the central role of polarized TCR recycling in this process, it appears to be also exploited for constitutive recycling (Liu et al., 2000; Onnis et al., 2016). In addition to the above-mentioned universal Rabs, several other Rabs have been identified in the TCR recycling pathway. These include Rab3d, Rab8a, Rab8b, Rab29, and Rab35 and its GAP EPI46C (Patino-Lopez et al., 2008; Soares et al., 2013a; Finetti et al., 2014, 2015b; Onnis et al., 2015). We have been able to show that some of these Rabs act sequentially in the pathway based on the ability of recycling TCRs to polarize to the IS (Figure 2). Of these, we have identified Rab8 as the most distal in the pathway, where it promotes the recruitment to endosomal TCRs of the v-SNARE VAMP3, which together with the t-SNAREs SNAP-23 and Syntaxin 4, that are recruited to the IS (Das et al., 2004), allows for the final fusion step to incorporate recycling TCRs into the plasma membrane (Finetti et al., 2015b).

**FIGURE 3:**
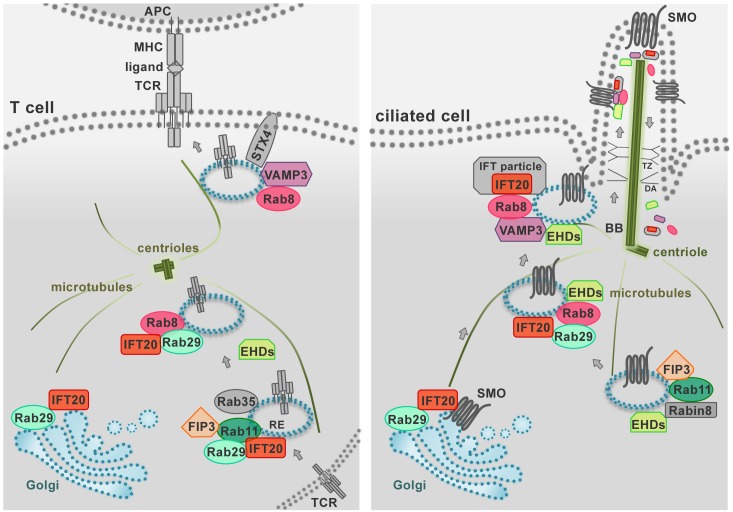
The endosomal trafficking pathways that control protein targeting to the primary cilium are co-opted by T cells for immune synapse assembly. The IS (left) represents the functional homolog of the primary cilium in the non-ciliated T cell (right) as these specialized structures share both structural properties and signaling pathways. During IS and primary cilium formation the centrioles and Golgi apparatus polarize beneath the respective signaling membrane domains. In addition, the polarized delivery of the T-cell receptor (TCR) and ciliary receptors (e.g., SMO, Smoothened) require common vesicular trafficking pathways coupling a recycling endosomal pool (marked by IFT20, Rab11, Rab29, FIP3) to the vesicle pool docked at the IS or at the primary cilium (marked by Rab8 and VAMP3). Notably, in ciliated cells IFT proteins shuttle cargo to the cilium and back to the cell body as large multimolecular complexes, known as IFT particles. In T cells, all the subunits of the IFT particles are expressed and IFT20 interacts with the IFT proteins IFT88, IFT57, and IFT52 (not shown) to promote TCR recycling to the IS. Similarly, a novel role of the EHD proteins in TCR trafficking has been recently described beyond their well-known function in ciliated cells. Shared traffic regulators are shown in color, other regulators in gray. BB, basal body; DA, distal appendages; RE, recycling endosome; TZ, transition zone.

A previously uncharacterized role has been recently ascribed to Eps15 homology domain (EHD) family proteins in constitutive TCR recycling to ensure optimal levels of surface TCR levels for subsequent T cell activation (Iseka et al., 2018). These proteins, that include EHD1-4, interact with Rab5, Rab11, Rab8a, and the respective effectors Rabenosyn-5, Rab11-FIP2 and MICAL-L1, regulating cargo recycling through the ERC (Naslavsky et al., 2006; Rahajeng et al., 2009; Sharma et al., 2009). Interestingly, EHD1 and EHD3 play a role in association with the Rab11–Rab8 cascade in early ciliogenesis (Lu et al., 2015; Figure 3). It is now well established that components of the machinery involved in ciliogenesis are also operational in the pathway of TCR recycling in T cells, which lack a primary cilium (Figure 3). This is exemplified by the intraflagellar transport (IFT) system component IFT20, which has been demonstrated to control TCR trafficking from EEs to REs together with other IFT components both during constitutive recycling and during polarized recycling to the IS upstream of Rab8 (Finetti et al., 2009, 2015b). Thus it is conceivable that EHD proteins may be also implicated in polarized TCR recycling in T cells.

In addition to the Rab GTPase network, both the tubulin and the actin cytoskeleton are central players in Rab11^+^ endosome recycling. REs move to the perinuclear ERC and back to the plasma membrane using microtubule tracks, and the forces for vesicle movement along microtubules are generated by the actin cytoskeleton, highlighting a potential key role of actin dynamics not only for TCR-MCs movement at the plasma membrane but also for polarized TCR recycling. Consistent with this notion, Zhang et al. (2017) have shown that Arpc2, a component of Arp2/3 actin-nucleating complex, is associated with TCR^+^ endosomes and that Arp2/3 complex-promoted actin polymerization is required for constitutive TCR^+^ endosome recycling. The Wiskott–Aldrich Syndrome Protein and SCAR Homolog WASH, a component of the retromer that controls retrograde traffic from EEs, couples Arp2/3 to endosomes through its partner FAM21, promoting the local nucleation of F-actin for force generation to allow for fission of vesicles carrying TCR cargo from EEs that have undergone retromer-mediated tubulation. WASH additionally interacts with tubulin, acting as a link between microtubule and actin cytoskeleton for endosome movement (Derivery et al., 2009; Gomez and Billadeau, 2009), thereby contributing to TCR recycling (Piotrowski et al., 2013; Figure 2). *De novo* actin polymerization during IS assembly is paralleled by *de novo* microtubule growth from the polarized centrosome, a process coordinated by microtubule plus-end binding protein EB1 which also controls the movement of endocytic TCRs to the IS by interacting with the CD3ζ ITAMs (Martín-Cófreces et al., 2012).

#### Lck and LAT Recycling

During IS assembly endocytic TCR trafficking intersects with the traffic routes of other receptors and signaling mediators to form intertwined signaling nanodomains at the plasma membrane, organized to promote a fully competent IS. Indeed, T cells exhibit distinct endosomal subpopulations that deliver to the IS TCRs, Lck and LAT with the assistance of specific sets of vesicular traffic regulators, including Rab GTPases, IFT proteins, and SNAREs (Das et al., 2004; Anton et al., 2008; Patino-Lopez et al., 2008; Finetti et al., 2009, 2015a; Martín-Cófreces et al., 2012; Larghi et al., 2013; Soares et al., 2013a).

Lck associates with Rab11^+^ endosomal compartments (Gorska et al., 2009; Soares et al., 2013b), and its transport to the IS and sorting to the cSMAC is regulated by the uncoordinated 119 protein (Unc119) (Gorska et al., 2009), the late endosomal transporter CD222 (Pfisterer et al., 2014), and the lipid raft-associated myelin and lymphocytes (MAL) protein (Anton et al., 2008, 2011). Interestingly, Unc119 assists the mobilization of Lck to endosomes that are recruited to the IS by extracting membrane-bound Lck through sequestration of its myristoyl group. These endosomes are subsequently released at the IS under the control of the small Arf-like GTPases Arl3 and Arl13b (Stephen et al., 2018). The fact that Unc119 and Arl3/Arl13b are central regulators of ciliogenesis (Wright et al., 2011) underscores the notion that the pathways that control ciliogenesis are exploited for IS assembly and function in the non-ciliated T cells, as also witnessed by the implication of the ciliary machinery in centrosome polarization and docking, actin clearance and signaling at the IS of cytotoxic T cells (de la Roche et al., 2013; Stinchcombe et al., 2015; Gawden-Bone et al., 2018). The endosomal localization of Lck, on which its function as regulator of TCR signaling depends, is mediated by the Rab11 effector and ciliogenesis regulator FIP3 (Rab11 family interacting protein-3) and, to a lesser extent, by its close homolog FIP4 (Horgan and McCaffrey, 2009; Wang and Deretic, 2015; Bouchet et al., 2017). FIP3 also indirectly modulates the surface levels of the TCR through the steady-state degradation of TCRζ that is constitutively phosphorylated by Lck (Bouchet et al., 2017). Interestingly, FIP3 also controls the ERC localization and IS targeting of another master regulator of endosomal recycling and cortical actin dynamics, namely the GTPase Rac1 (Bouchet et al., 2016). The FIP3-dependent localization of Lck and Rac1 in Rab11^+^ vesicles underscores the importance of the interplay between endocytic trafficking and the cytoskeleton in ensuring the synaptic delivery of crucial components of the TCR signaling cascade (Bouchet et al., 2018).

Consistent with a mechanism involving the combination of specific components of the cellular trafficking machinery to achieve specificity in the polarized transport of different cargoes, the intracellular pool of LAT is mobilized to the IS in endosomes marked by Rab27 and Rab37 which are delivered to the IS with the assistance of IFT20 and the v-SNARE VAMP-7 in a process regulated by Lck-dependent Ca^2+^ mobilization and the Ca^2+^ sensor synaptotagmin-7 (Purbhoo et al., 2010; Larghi et al., 2013; Soares et al., 2013b; Vivar et al., 2016). There phosphorylated LAT undergoes c-Cbl-mediated ubiquitylation and clathrin-dependent endocytosis (Brignatz et al., 2005; Balagopalan et al., 2011). Of note, TCR-dependent signaling was shown to be enhanced in T cells expressing ubiquilytation-resistant mutants of LAT, highlighting LAT ubiquitylation as a mechanism of attenuation of T cell signaling (Balagopalan et al., 2011). In a recent report, Carpier et al. (2018) showed that, after internalization at the plasma membrane, LAT transits through the Golgi-TGN via Rab6 and the t-SNARE Syntaxin-16, which regulate retrograde endosome-to-Golgi/TGN retrograde transport. This process is essential for the polarization and delivery of endosome-associated LAT to the IS (Carpier et al., 2018; Figure 2).

The relative contribution of plasma membrane and endosomal LAT to TCR signaling is as yet debated. While the recruitment of vesicular LAT is delayed compared to surface LAT (Bonello et al., 2004), Larghi et al. (2013) have provided evidence that LAT phosphorylation and assembly of the LAT signalosome is dependent on the VAMP-7-dependent recruitment of vesicular LAT to endosomes do not fuse with the plasma membrane but remain associated with subsynaptic vesicles together with VAMP-7. At variance, Balagopalan et al. (2018) proposed a model, based on high-resolution lattice-sheet microscopy on live cells, whereby cell surface LAT is rapidly recruited to and phoshorylated at TCR activation sites, with the endosomal pool being recruited at a later time to interact with signaling microclusters.

While the mechanisms controlling endocytic trafficking during IS assembly need to be fully elucidated, the existence of tailor-made trafficking pathways for different signaling molecules underscores the complexity of regulatory networks that must be tightly coordinated in space and time to control T cell activation.

### Dual Role of TCR Endocytosis in Signal Termination and Sustained Signaling

That TCR endocytosis is an important mechanism to terminate signaling is strongly supported by the finding that TCR-MCs that fail to be internalized in TSG101-deficient T cells remain signaling-competent, resulting in T cell hyperactivation (Vardhana et al., 2010). However, recent evidence supports the existence of a signaling TCR endosome in T cells, as initially exemplified by the EGF receptor which continues to signal after its ligand-induced internalization (Bakker et al., 2017). Using fluorescent reporters suitable for live cell imaging, Yudushkin and Vale (2010) showed that plasma membrane-derived, tyrosine-phosphorylated CD3ζ accumulates in perinuclear endosomes in activated T cells. Interestingly, active Lck was also found associated with these endosomes, suggesting that endosomal Lck could contribute to sustain CD3ζ phosphorylation after its internalization. Consistent with this notion, Willinger et al. (2015) showed that the T-cell-specific deletion of dynamin 2, an essential component of the endocytic machinery that is required for TCR internalization, results in impaired TCR signaling, concomitant with impaired homeostatic proliferation as well as clonal expansion *in vivo*, indicating that endocytosis sustains TCR signaling. The RhoG- and TC21-dependent phagocytosis of TCRs still engaged with their pMHC (see section “TCR Endocytosis”) may represent another means to sustain endosomal signaling of internalized receptors. In support of this notion, upregulation of early activation markers was found to be impaired in RhoG- or TC21-deficient T cells (Martínez-Martín et al., 2011).

Interestingly, del Río-Iñiguez et al. (2018) have recently shown that HIV-1 infection of T cells alters endosomal traffic and actin cytoskeleton remodeling, ultimately impacting on their activation. This function is mediated by the virulence factor Nef, which promotes the sequestration of Lck and Rac1 in a pericentrosomal compartment that is enriched in tyrosine-phosphorylated CD3ζ as well as ZAP-70, SLP-76 and the actin regulator Vav1, but selectively lacks LAT. Hence signaling endosomes emerge as targets for immune subversion during host-pathogen interactions.

### Exocytic Trafficking at the IS for Intercellular Communication

While the cSMAC recruitment of ubiquitin ligases and components of the endocytic degradation machinery highlights the cSMAC as the IS domain where the TCR complex is endocytosed, a new twist to the story has emerged with the finding, based on ultrastructural analyses, that the cSMAC is also the site of release of plasma membrane-derived, TCR-enriched extracellular microvesicles (ectosomes) that bud from the IS center and are taken up by the APC (Vardhana et al., 2010; Choudhuri et al., 2014). The IS is also the site of release of canonical, MVB-derived CD63^+^ exosomes that are enriched in miRNAs and are unidirectionally transferred to and modulate gene expression in the APC (Mittelbrunn et al., 2011). These findings highlight a central role for the IS not only in elaborating signals provided by the APC for activation but also in providing in turn information carried by extracellular vesicles to modulate APC function.

## Future Perspectives

From the initial scenario of the TCR as a receptor undergoing constitutive recycling in quiescent cells to allow for multiple opportunities of quality control during its long lifetime, or alternatively being targeted for degradation following ligand-induced internalization, a much more complex scenario has emerged, where the fate of a TCR is not limited to recycling or degradation, but extends to signaling from endosomes and to becoming incorporated into extracellular vesicles with the ability to affect the function of bystander cognate APCs. These alternative fates are not necessarily mutually exclusive. For example, internalized TCRs may undergo recycling or remain associated with signaling endosomes before being eventually targeted for degradation. What we currently know about how the signals triggered at the IS can couple the TCR to the different endocytic pathways that control these alternative fates is as yet very fragmentary. The affinity of the ligand and hence the strength of the signal may be one of the determinants in the preferential mobilization of the specific trafficking pathway that will lead to the appropriate fate of engaged TCR, as strikingly exemplified by the association of TCR-MCs with TSG101 and their subsequent endocytosis following engagement by strong ligands, but not by weak ligands (Vardhana et al., 2010). In this respect, considering the impact of signal strength on CD4^+^ T cell polarization to specific helper subsets (van Panhuys, 2016), it will be important to analyze endocytic traffic at the IS in T cell primed in the presence of polarizing cytokines.

Another emerging question is the cooperation between plasma membrane-associated TCRs and signaling mediators and their endosomal counterparts in the spatiotemporal control of IS signaling. For example, while endosomal Lck must be delivered to the IS membrane for signaling to occur, it has been suggested to also sustain the phosphorylation of endosomal TCRs following their ligand-induced internalization, implicating that outgoing Lck^+^ endosomes and incoming pTCRζ^+^ endosomes might functionally intersect during the respective journeys (Yudushkin and Vale, 2010). A similar scenario has been proposed for endosomal LAT, which has been shown to remain associated with subsynaptic vesicles wherefrom it has been hypothesized to either become *trans*-phosphorylated by the plasma membrane TCR-associated kinases to assemble the LAT signalosome or *cis*-phosphorylated after fusion between endosomes containing internalized kinase-associated TCR complexes and LAT-containing vesicles (Larghi et al., 2013). Although this model must be reconciled with the alternative model positing that plasma membrane-associated LAT phosphorylated at TCR engagement sites precedes signaling by endosome-associated LAT and that fusion of LAT^+^ vesicles with the synaptic membrane may be required for TCR signal amplification (Balagopalan et al., 2018), it casts an interesting light on endosomes as signaling hubs that could participate in signal amplification and allow for signaling to continue after engaged TCRs have been internalized. The study of the composition of these endosomes may shed light on whether the associated signaling complexes are actually similar to the ones assembled at activation sites at the plasma membrane or represent distinct complexes that contribute to signal diversification.

Interestingly, while Rab11^+^ endosomes are used as universal carriers for the transport of the intracellular pools of TCR and signaling mediators to the IS, specifically tailored pathways control the traffic of individual cargoes. For example, we have reported that the TCR, the transferrin receptor and the chemokine receptor CXCR4 use a specific combination of IFT and Rab proteins for recycling (Finetti et al., 2009, 2014, 2015a; Onnis et al., 2015, 2016). Additionally, Lck, TCRζ and LAT are associated with endosome subpopulations marked by specific sets of Rab GTPases, adaptors and SNAREs and differentially regulated by Ca^2+^ for synaptic release (Das et al., 2004; Anton et al., 2008; Patino-Lopez et al., 2008; Finetti et al., 2009, 2015a; Larghi et al., 2013; Soares et al., 2013a). How this mosaic of endosomes, each carrying a vital part of the TCR signaling machinery, is spatiotemporally coordinated is a major open question.

While vesicular trafficking has long been known to be central to the effector functions of both helper and cytotoxic effector T cells, the emerging evidence that it is also exploited for intercellular communication highlights a new priority area of research in the T cell field. For example, in addition to transferring microvesicle-associated miRNAs and TCRs to APCs (Mittelbrunn et al., 2011; Choudhuri et al., 2014), T cells have been reported to transfer CD40L to B cells during T cell help. While the underlying mechanism remains to be identified, this process has been shown to correlate with B cell activation, suggesting the possibility that sustained CD40 signaling after co-internalization with T cell-derived CD40L may allow for sustained signaling, enabling survival of B cells following their interaction with helper T cells in the germinal centers (Gardell and Parker, 2017). However, CD4^+^ derived T cell exosomes can inhibit CD4^+^ T cell proliferation and CD8^+^ CTL responses, similar to Treg-derived exosomes, and moreover some evidence suggests that T cell exosomes may be implicated in tumor progression and invasion by targeting tumor cells as well as endothelial cells (reviewed in Lu et al., 2018). Hence characterizing the extracellular T cells and the endocytic pathways that control their generation and release will not only provide important insight into the mechanisms of target cell instruction by T cells but may also provide new opportunities for cellular strategies of therapeutic intervention.

Finally, an imbalance in the endocytic events that control TCR recycling and degradation has been identified as an important determinant in immune-related diseases. Abnormalities in TCR and CD4 recycling have been associated with the critical loss of TCR and the resulting impairment in IL-2 production in systemic lupus erythematosus T cells (Fernandez et al., 2009; Telarico and Perl, 2012). Additionally, AKAP9-dependent TCR recycling has been recently shown to be essential for effector T cell re-activation and retention in tissues, and abnormalities in these processes have been associated with disease in experimental models of multiple sclerosis and crescentic glomerulonephritis (Herter et al., 2015). At a more general level, alterations in endocytic trafficking have been associated with a variety of diseases, cancer being one of the major categories. This is exemplified by B-cell chronic lymphocytic leukemia, where enhanced recycling maintains abnormally high the surface levels of the homing receptors CXCR4 and CCR7, favoring the retention of leukemic cells in the pro-survival and protective lymphoid niche (Patrussi et al., 2015). Hence a better understanding of the endocytic pathways that control IS assembly and function is expected to bring to light new candidates for the therapeutic modulation of IS function in immune dysregulation.

## Author Contributions

All authors listed have made a substantial, direct and intellectual contribution to the work, and approved it for publication. AO and CB wrote the manuscript. AO prepared the figures.

## Conflict of Interest Statement

The authors declare that the research was conducted in the absence of any commercial or financial relationships that could be construed as a potential conflict of interest.
